# Lignin–Carbohydrate Nano-Sized Structures: An Evidence of Intracellular Lignin Biosynthesis?

**DOI:** 10.3390/plants15030399

**Published:** 2026-01-28

**Authors:** Nikita A. Shutskiy, Sergey A. Pokryshkin, Elena A. Anikeenko, Anna V. Faleva, Artyom V. Belesov, Ilya I. Pikovskoi, Ksenia S. Vashukova, Ludmila V. Mayer, Dmitry S. Kosyakov, Maria S. Kalmykova, Dmitry G. Chukhchin

**Affiliations:** 1 Department of Biology, Ecology and Biotechnology, Core Facility Center «Arktika», Northern (Arctic) Federal University, Northern Dvina Embankment 17, Arkhangelsk 163000, Russia; nikitashutskijj@rambler.ru (N.A.S.); s.pokryshkin@narfu.ru (S.A.P.); a.anikeenko@narfu.ru (E.A.A.); a.belesov@narfu.ru (A.V.B.); i.pikovskoj@narfu.ru (I.I.P.); k.bolotova@narfu.ru (K.S.V.); d.kosyakov@narfu.ru (D.S.K.); kalmykova.m@edu.narfu.ru (M.S.K.); dimatsch@mail.ru (D.G.C.); 2 Department of Histology, Cytology and Embryology, Northern State Medical University, Troitsky Ave. 51, Arkhangelsk 163000, Russia; 3 Department of General and Bioorganic Chemistry, Northern State Medical University, Troitsky Ave. 51, Arkhangelsk 163000, Russia; mayer58@mail.ru

**Keywords:** ultrastructure of wood, cell wall, cellulose microfibrils, lignin–carbohydrate complex

## Abstract

The spatial localization of plant secondary cell wall polymers is a controversial issue. A relief of parallel-organized cellulose microfibrils was discovered, on the surface of which spherical nanoparticles were visualized. Spherical nanoparticles with a diameter of 20–50 nm were isolated using size exclusion chromatography from an aqueous extract of differentiating xylem of Norway spruce and visualized by SEM and AFM. The composition of isolated nanoparticles was determined by pyrolytic GC-MS, ^1^H NMR spectroscopy, and nitrobenzene oxidation, followed by separation of the products by liquid chromatography. Lignin was detected in the isolated nanoparticles already at the stage of cell wall formation. The hypothesis about the intracellular synthesis of lignin was proposed based on the results obtained. Lignin in the form of a lignocarbohydrate complex is formed not in the cell wall, but inside the cell. The formation of lignin–carbohydrate complexes occurs in Golgi apparatus and vesicles, which discharged into the inner surface of the cell wall simultaneously with the deposition of cellulose microfibrils. A new model of the structure of secondary cell wall postulates the formation of cellulose microfibrils surrounded by lignin–carbohydrate spherical complexes having a carbohydrate shell and an aromatic core.

## 1. Introduction

The lignin–carbohydrate matrix of cell wall in plants is a superposition of interpenetrating networks formed by hydrogen, carbon–carbon, ether bonds of lignin, and lignin–carbohydrate bonds [1,2,3,4,5]. Additional strength of the composition is given by the mechanical linkages of macromolecular segments of lignin and hemicellulose, both among themselves and with cellulose [6,7].

Currently, several models of the secondary cell wall have been proposed. Using 3D modeling, Jakes et al. provided a variant where cellulose fibers are arranged almost parallel and covered with hemicelluloses, whereas unstructured lignin surrounds the fibers [8]. Erfani et al. believe that lignin acts as a filler between cellulose and hemicelluloses and serves to hold the lignocellulosic matrix together; interactions of lignin units with xylan are observed but the direct interactions of cellulose with lignin are less dominant [9]. However, the quantitative ratio of cellulose, hemicellulose and lignin in the cell wall is poorly taken into account in these graphic models.

According to most modern concepts, lignin is deposited in the apoplast after the formation of a secondary cell wall and cell death. Cell plate formation in plant cells is a multistep process. Plant lignification is occurring in three stages: biosynthesis of lignin precursors, monolignols, in the cell cytosol, transport of monolignols to the cell wall, and dehydrogenative polymerization of monolignols within the cell wall [10].

For monolignol transport through the plasma membrane, either as free monolignols or as glucosides, one can envisage four possible mechanisms: passive diffusion through the plasma membrane based on hydrophobic interactions between the monolignols and membrane lipids; Golgi-vesicle mediated transport; active transport via plasma membrane transport proteins; facilitated diffusion through plasma membrane channels [11]. Data of Kaneda et al. [12] are more consistent with a transporter-mediated export of monolignols than a Golgi-mediated export hypothesis; however, a vacuolar localization of monolignol glucosides has been speculated previously [13,14].

The lignin precursors are exported into the cell wall where localized oxidative enzymes generate radicals that then facilitate the polymerization of lignin [11]. The oxidative enzymes in the cell wall continue to function even after programmed cell death [15].

The existing model assumes that lignin polymerizes in apoplast as a free radical spontaneous process, but it cannot explain how the complex LCC structure can be precisely generated in the cell wall without the assistance of enzymes and energy. The theoretical possibility of extracellular lignin synthesis does not mean that lignin is synthesized outside the cell. The concept of extracellular lignin synthesis has a number of contradictions:

1. Firstly, motion and activity of large lignin-synthesizing enzymes and lignin precursors inside the tight cell wall fixed by hydrogen bonds are doubtful;
2. Secondly, formation of chemical bonds between lignin and hemicelluloses requires energy and macroergic molecules (ATP for example); formation of these bonds in cell wall is unlikely. Spontaneous formation of lignin–hemicelluloses bonds in vitro has not been observed;
3. Thirdly, non-cellulose polymers in the cell wall are visualized as spherical nanoparticles of 20–50 nm [2]. In the above cell wall models, the polymers are spatially arranged in a different way.

In contrast to the common belief that monolignols combinatorial coupling is restricted to the apoplast, a metabolic pathway was proposed, involving intracellular combinatorial coupling of monolignol radicals, followed by oligomer glycosylation and vacuolar transport [16]. It is also known that biosynthesis of the arabinoxylan backbone and arabinose take place intracellularly in the Golgi vesicles [17,18]. The structural polysaccharides and pectin are synthesized in the Golgi and transported via Golgi derived vesicles to the apoplast [19]. Subsequently, cell wall polysaccharides, associated enzymes, and glycoproteins are carried to specific cell wall deposition sites by vesicle transport pathways, which remain poorly resolved.

In plant cells, the Golgi apparatus is divided into numerous (up to several hundred per cell) Golgi complexes, called dictyosomes and distributed as small stacks in the cytoplasm [20]. Golgi vesicles can vary widely in size [21,22]. The individual cell plate-forming vesicles are uniformly small in diameter (64 nm ± 12 nm) [23].

According to our proposals, lignin like other non-cellulose polymers of the cell wall is synthesized in the cisternae of Golgi apparatus and transported to the cell wall by vesicles derived from the Golgi stacks.

The aim of this work is to propose a hypothesis of intracellular lignin synthesis and new model of the secondary cell wall in plants. To prove this, it is necessary to isolate the water-soluble particles of the Golgi vesicles from the differentiating xylem and detect lignin in them.

## 2. Results

### 2.1. SEM and AFM Visualization and Isolation of the Nanoparticles

**Figure 1:**
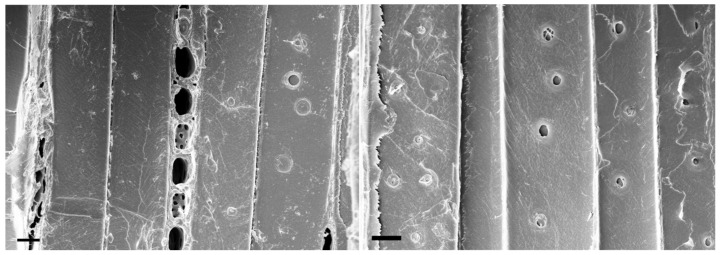
SEM images from opposite sides of the slice, demonstrating that the xylem in the samples is in the differentiating stage. Bars 10 µm.

To assess the degree of differentiation of the xylem of the isolated samples, one can use the morphology of the bordered pores of the tracheids [24] and the direction of the cellulose microfibrils in the secondary cell wall of the tracheids on the inner surface. SEM images of the slices were obtained (Figure 1).

The “immature” pores (small size, no border) of the tracheids are observed on both sides of the slice. Mature xylem does not have such objects. In addition, there are no microfibrils with a direction parallel to the fiber axis (S2). Thus, the isolated samples represent differentiating xylem during the formation of the S1 layer of the cell wall.

**Figure 2:**
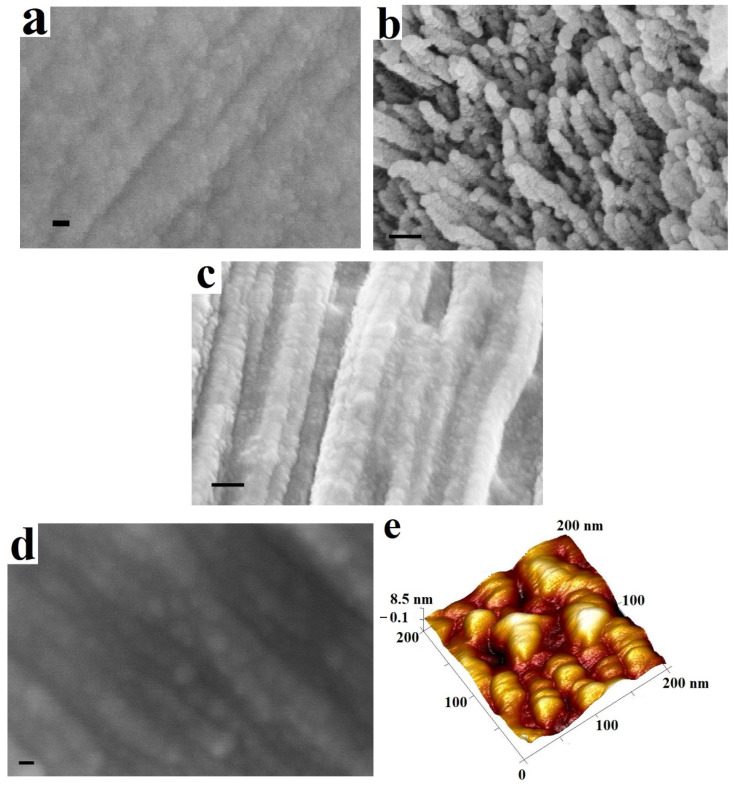
SEM-image of longitudinal split of S2 layer of balsam poplar (**a**), transverse split of wheat S2 layer where spherical particles are visible on broken cellulose microfibrils (**b**); SEM-images (**c**,**d**) and AFM-image (**e**) of the S1 layer split of spruce cell wall. Bars 20 nm (**a**), 100 nm (**b**), 100 nm (**c**), 20 nm (**d**).

SEM and AFM studies of cell wall splits visualized a parallel-laid microfibrils relief, on the surface of which there are spherical nanoparticles with a diameter of 20–30 nm (Figure 2).

A similar picture is observed for gymnosperms and angiosperms, monocots and dicots in the S1 and S2 layers of the secondary cell wall (Figure 2a–d). The non-cellulose components of the cell walls are unevenly distributed and localized in these particles. This pattern was also noted earlier on the example of juniper [2] (Figure 2b). AFM studies were used as an alternative tool for visualizing microfibrils with spherical nanoparticles on their surfaces (Figure 2e). The parts of cellulose microfibrils that lacked nanoparticles on their surfaces were identified using AFM. The mechanical properties of these particles differ significantly from those of cellulose microfibrils. The DMT modulus of spherical particles is less than 1 GPa, while that of cellulose microfibrils is greater than 3 GPa. Furthermore, differences in the adhesion coefficient of the cantilever to the surface were detected: the coefficient was 30–40 nN for spherical particles, while it was 5–10 nN for microfibrils.

**Figure 3:**
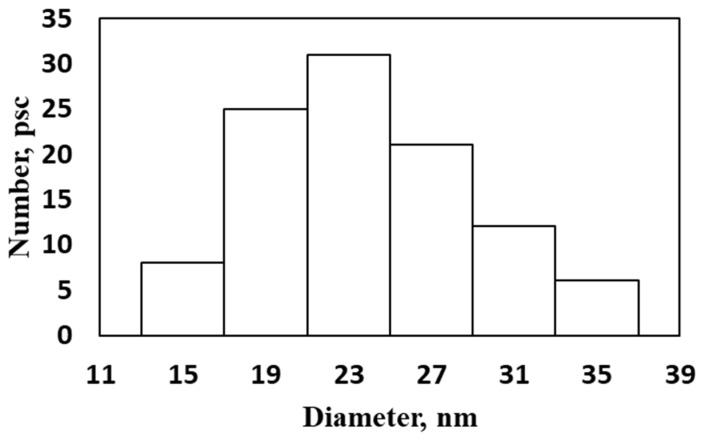
Size distribution of spherical nanoparticles on S1 surface of spruce splits.

The size distribution of the nanoparticles on spruce S1 layer was determined (Figure 3). The average nanoparticle size was 24.0 ± 1.03 nm with probability 0.95.

The spherical particles visualized in Figure 2 and Figure 3 are likely delivered to the surface of cellulose microfibrils during cell wall formation. Considering the composition of the secondary plant cell wall, these are particles of non-cellulosic nature, which may contain lignin and hemicelluloses. The particle sizes at this stage of the study suggest the involvement of Golgi vesicles in their transport. This is also reasonable, since the Golgi complex is responsible for the synthesis of non-cellulosic polysaccharides. Further, the study of water-soluble particles of the Golgi vesicles from the differentiating xylem and detect lignin in them was continued on spruce samples. It is believed that lignin is water-insoluble. We believe that lignin in the form of lignin–carbohydrate complexes can be dissolved in water. Lignin presence in aqueous extract of the differentiating xylem specifically in the fraction of the particles comparable to Golgi vesicles (with or without of phospholipid shell) allows us to make an assumption about intracellular lignin synthesis. Intracellular lignin–carbohydrate complexes in Golgi vesicles as well as on the surface of cell wall fragments can be isolated during extraction and fractionation.

**Figure 4:**
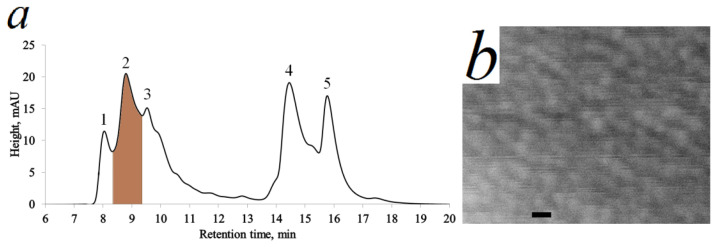
HPLC-SEC chromatogram of aqueous extract of differentiating spruce xylem. Peak of fraction with particle sizes from 20 to 50 nm (8.3–9.3 min) marked with brown (**a**). SEM-image of spherical nanoparticles obtained from second isolated fraction corresponding to peak 2 (**b**). Bars 30 nm.

The aqueous xylem extract was fractionated using HPLC-SEC after RNA hydrolysis (ribosomes destruction). Different retention time corresponded to the different particle fractions depending on their size. The BioSep-SEC-S4000 preparative column with the first fraction isolates particles with size ≥ 50 nm (peak 1), the particles range in the second fraction is 20–50 nm (peak 2), fractions three to five include particles less than 20 nm—peaks 3–5 (Figure 4a).

The SEM-image of the nanoparticles from fraction corresponding to the peak 2 (8.3–9.3 min) is shown in Figure 4b. The particle sizes of the second isolated fraction (peak 2, Figure 4a) correlate with the particle sizes in Figure 2.

The isolated fraction corresponding to the peak 2 (Figure 4a) was washed with distilled water in Microcon YM-100 concentrators (Amicon™ Bioseparations) with pore diameter 100 kDa. Thus, low-molecular components of the eluent with sizes less than 100 kDa were removed from the fraction. This washed fraction was used in further analysis for lignin detection in nanoparticles from aqueous extract of the differentiating xylem.

The mass of these spherical nanoparticles (Figure 4b) is more than 4 MDa considering the particle sizes (about 20 nm) at density of about 1500 kg/m^3^.

Extraction from mature xylem and chromatographic separation were carried out as well. Extraction, chromatographic, and detection conditions were kept identical between the differentiating and mature xylem samples. The value corresponding to the peak 2 was 21 arb. units in differentiating xylem. All other things being equal, the optical density in the region of the 2nd fraction in mature xylem is more than 100 times lower (0.2 arb. units). Thus, particles of the lignocarbohydrate complex are extracted only from living cells of differentiating xylem.

### 2.2. The Hypothesis Verification: Lignin Detection in Nanoparticles from Aqueous Extract of the Differentiating Xylem

**Figure 5:**
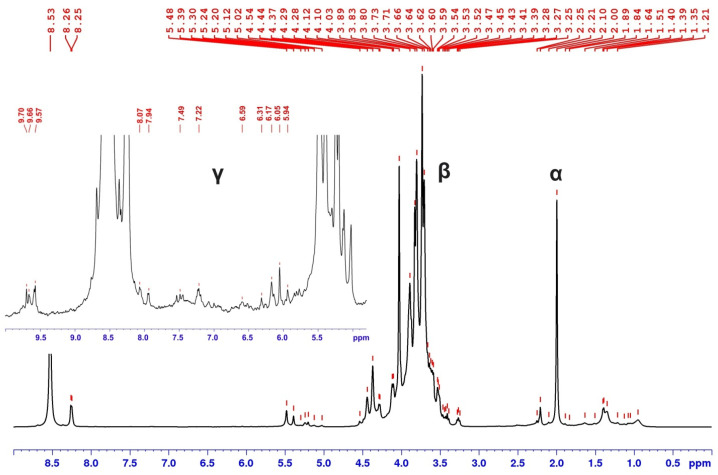
^1^H NMR spectrum of fraction from spruce xylem extract: α—signal of acetate protons (2.0 ppm); β—signals of protons in composition of polysaccharides (3.0–4.4/5.0–5.5 ppm); γ—a local magnification region of signals at 5.5–8.0 ppm generated by protons of aromatic structures.

The results of analysis of particles from isolated fraction by ^1^H NMR spectroscopy show that the main signals observed on the ^1^H NMR spectrum corresponding to the carbohydrate component (Figure 5).

The signals of aromatic structures are observed at a sufficiently low intensity level in the area of δH 5.5–8.0 ppm. The values of the chemical shifts of the signals observed in this area correspond to the values of the chemical shift of H- and G-type lignin models described in the literature [25]; Protons of H-units generate resonances in the range δH (ppm) ~7.3–7.1 at C_2_-H,C_6_-H and ~6.9–6.6 at C_3_-H,C_5_-H, whereas the observed signals in the range ~7.0–6.9 from C_2_-H, C_6_-H and ~6.9–6.70 from C_5_-H are responsible for the presence of G-type structures. The low intensity of the lignin protons signals relative to the those for carbohydrates is typical and is associated with the heterogeneity of the polymer units.

**Figure 6:**
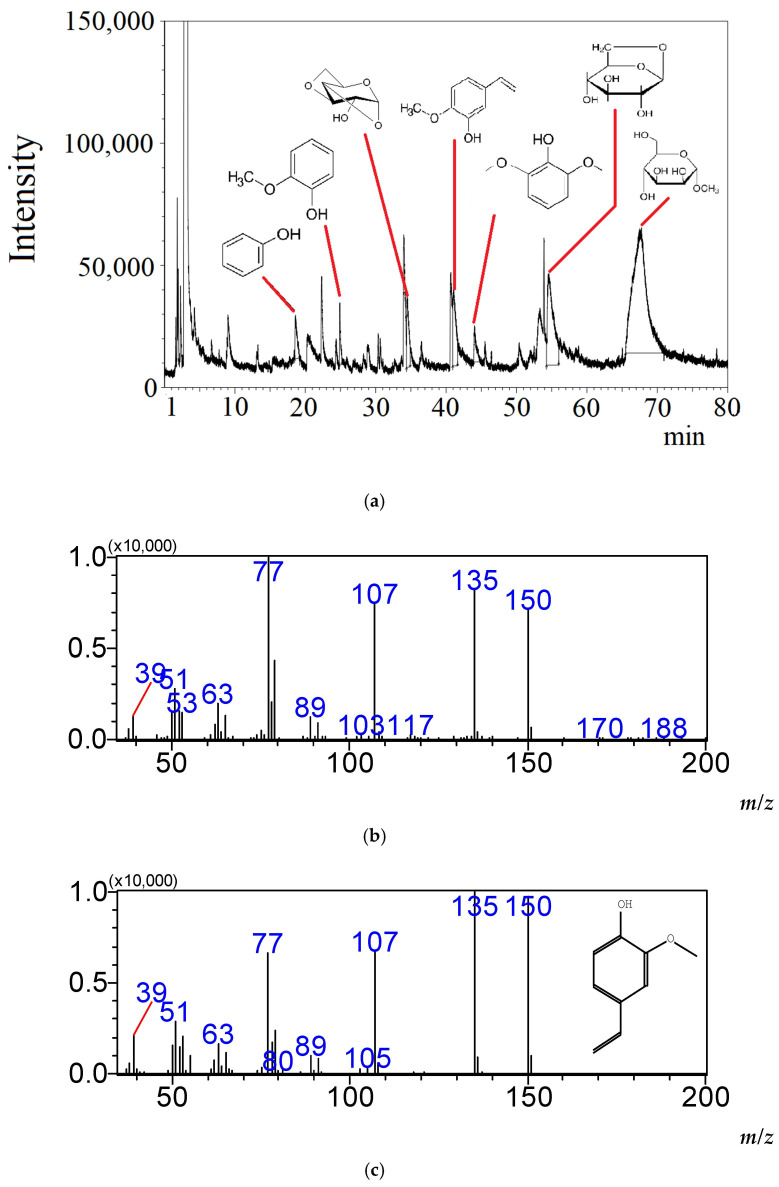
Pyrolysis products of isolated fraction and example of identification thereof. Chromatogram of pyrolysis products of isolated fraction: phenol; phenol, 2-methoxy (Guaiacol); 1,4:3,6-Dianhydro-α-D-glucopyranose; 2-Methoxy-4-vinylphenol (4-vinylguaiacol); syringol; β-D-Glucopyranose, 1,6-anhydro-; α-Dmannofuranoside, methyl (similarity index from 89 to 93) (**a**). Mass spectrum of decomposition product corresponding to chromatogram peak at 41 min in Figure 6a (**b**). Mass spectrum of degradation products of vinyl guaiacol from library search (similarity index 90) (**c**).

The Py-GC-MS analysis was performed to verification the assumption about the presence of lignin polymer structures in nanoparticles. The decomposition products such as phenol, guaiacol and vinyl guaiacol was detected (Figure 6) during pyrolysis of the isolated fraction.

The results give grounds to speak about the presence of lignin in the nanoparticles of the isolated fraction. Glucopyranose, methyl-mannofuranoside, etc. suggest the presence of polysaccharides in the fraction as well. The mentioned components were found only in trace amounts in the pyrolysis products of the fractions corresponding to the peaks 1 and 3 in Figure 4a.

According to the current theory of lignin synthesis, for example [6], there should be intracellular vesicles that could correspond to the particle size of the second fraction and contain monolignols. Wout Boerjan’s group has previously shown that oligolignols can accumulate in significant proportions in the vacuole or cytosolic compartments [16]. Therefore, the aromatic substances presented in Figure 6 can theoretically be formed during the pyrolysis of such (intravesicular) monolignols, some lignans and oligolignols.

The second fraction was used to prove that aromatic products in pyrolytic GC-MS are formed precisely from polymerized lignin (lignin–carbohydrate complexes). Sodium dodecyl sulfate (SDS) was added to the second fraction to a concentration of 0.5% and kept for 24 h. SDS destroys all kinds of phospholipid vesicles and membranes. Size exclusion HPLC was performed for the second fraction without vesicles. The peak maximum naturally shifted by 0.7 min to the right. Nevertheless, the mass of such substances amounts to millions of Daltons. This excludes the presence of monolignols, oligolignols and lignans. The contents of the peak substances were analyzed by Py-GC-MS. The same substances as shown in Figure 6 were obtained. Thus, it has been proven that the pyrolysis products presented in Figure 6 are obtained directly from lignin–carbohydrate complexes containing lignin in the form of a polymer.

As a result of chromatographic analysis of products of nitrobenzene oxidation it was also shown that 4-hydroxybenzaldehyde and vanillin were detected in a ratio of 2/1 in the particles from isolated fraction (Table 1).

## 3. Discussion

The synthesis of polysaccharides from monosaccharides occurs by using additional energy. The intracellular macroergic substances are used, for example, ATP and uridine-diphosphate (UDP). Therefore, the synthesis of hemicelluloses outside the cell is impossible [26]. Cellulose is synthesized by rosettes on the cell membrane from UDP-glucose. The plant Golgi apparatus has an important role in glycosylation and sorting, but is also a major biosynthetic organelle that synthesizes large quantities of cell wall polysaccharides. Hemicelluloses and pectin substances are synthesized in the Golgi apparatus and discharged into the cell wall from Golgi vesicles [21,27].

However, for lignin, the hypothesis of extracellular synthesis has become more widespread despite many contradictions. There is a high concentration of glycosides of phenylpropane units [28] in cells synthesizing lignin. The synthesis of lignin involves the enzymatic removal of glucose from syringin and coniferin, the emission of lignin precursors from the cell, the dehydrogenase action towards the phenylpropane units, radical formation and their subsequent random combination [29]. Thus, extracellular synthesis requires the emission of lignin monomers and enzymes from the cell, as well as the presence of extracellular oxidizer (O_2_ or H_2_O_2_) receiving hydrogen from dehydrogenases. In this case, it is unclear how the lignin-hemicellulose chemical bond is formed, because the process requires energy.

No one has observed the formation of lignin-hemicelluloses bonds in vitro. Many scientists, for example, Terashima et al. [30] have tried to obtain in vitro from coniferyl alcohol lignins are similar in structure to lignins of milled wood lignins (MWL). However, the ^13^C-NMR data showed significant differences between these lignins in the types of chemical bonds formed between the units and their ratio.

The coniferin bond of aromatics with a carbohydrate is formed only inside the cell. Extracellular energy (for example, in the form of ATP) is an unlikely event and damage-associated signal in plants [31].

It is noted [32,33] that lignin located in the cell wall is heterogeneous both in the structure of the macromolecule and in the ratio of syringyl (S), guaiacyl (G), and p-hydroxyphenylpropane (H) units. This means that the lignin polymerization is carried out during the formation of the cell wall. The concept of lignification after cell death is questionable since low-molecular-weight lignin precursors, diffusing into the cell wall, would erase the gradients of lignin structures. If a dense cell wall hinders the diffusion of lignin precursors, then it makes the enzymatic process of their polymerization impossible.

A very wide distribution of lignin–carbohydrate complex by molecular weight should be obtained during the grinding of cell walls (based on the concept of extracellular lignin synthesis). The water-soluble fraction at pH 5 may contain only a low-molecular component. According to pyrolytic GC-MS, fragments of lignin and polysaccharides were found only in the isolated fraction with a particle diameter comparable to the diameter of secreted Golgi vesicles. This fraction may contain both lignin–carbohydrate complexes detached from the surface of the cell walls, and intracellular lignin–carbohydrate complexes in the phospholipid shell. The solubility of the particles with a mass of several million Daltons in an acetate buffer at pH 5 indicates that only hydrophilic substances (the carbohydrate part of lignin–carbohydrate complex or the polar part of phospholipids) can be on their surface.

**Figure 7:**
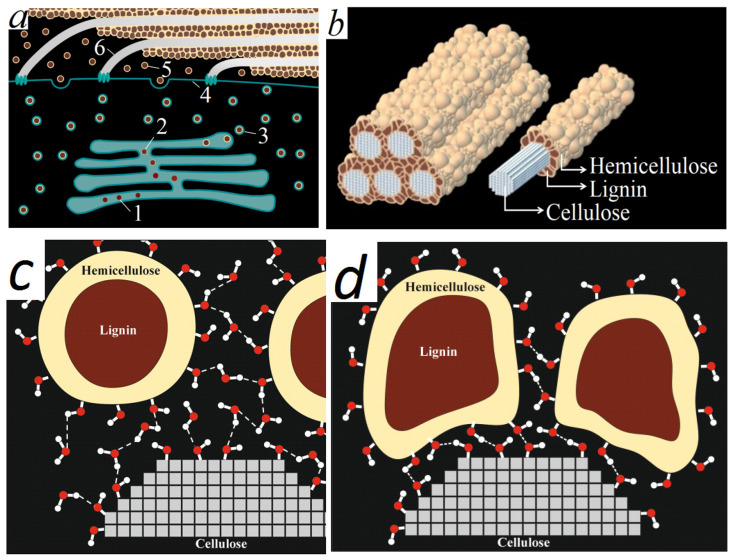
Formation of lignin–carbohydrate complexes in Golgi apparatus and deposition of secondary cell wall (**a**): 1—lignin formed in Golgi apparatus; 2—emerging lignin–carbohydrate complex; 3—vesicles of Golgi apparatus with a lignin–carbohydrate complex; 4—plasma membrane; 5—lignin–carbohydrate complex; 6—cellulose microfibril. A new model of structure of secondary cell wall (**b**). Model of secondary cell wall for differentiating xylem (**c**). Model of secondary cell wall for mature xylem (**d**).

The hypothesis of intracellular lignin synthesis allows the use the energy of hydrogen split off by dehydrogenases, transferring it, for example, to nicotinamide adenine dinucleotide (NAD) and further along the respiratory chain to obtain ATP. With this path there is no need for extracellular dehydrogenases and enzymes forming a lignin–carbohydrate bond. The lignin–carbohydrate complex will be synthesized in the Golgi stacks and discharged using vesicles into the inner surface of the cell wall in parallel with the laying of cellulose microfibrils (Figure 7a).

The presence of lignin-synthesizing enzymes in the cell wall can be explained not by the place of their action, but by transportation together with the lignin from the cell in Golgi vesicles.

We can assume the following mechanism: a micelle containing lignin structures inside and carbohydrate structures outside is formed in the Golgi apparatus. First, glycosides such as coniferin or syringin form a micelle, and then their hydrolysis, polymerization, and polycondensation take place to form lignin–carbohydrate complexes. Both hydrolysis of glycosides and the formation of new lignin–carbohydrate bonds are possible. The sequential synthesis of lignin and then hemicellulose provides the formation of a water-soluble structure. The presence of carbohydrates on the outside allows the lignin–carbohydrate complex to obtain a hydrophilic surface and affinity for cellulose microfibrils of the cell wall.

The surface of spherical nanoparticles contains a large number of OH groups. Spherical nanoparticles acquire a negative potential upon entering the apoplast as a result of Brownian motion. Therefore, the nanoparticles are stable in the dissolved state. The nanoparticles lose their motion upon reaching the inner surface of the cell wall and are attached to the surface via H-bonds (Figure 7c). Hemicelluloses form a strong bond with the cellulose microfibrils due to hydrogen bonds between a large number of OH groups of cellulose and hemicellulose (Figure 7c,d).

Sometimes the composition of the intercellular substance is given as an argument in favor of the extracellular synthesis of lignin; it is noted [32] that p-hydroxyphenyl units in pine are formed in the middle plates and in the corners of the cell wall at early stage of the lignifications. We believe that the presence of lignin in these locations can be explained by the fact that osmotic pressure increases in the cell before the formation of the secondary cell wall; the cell spreads out as much as possible and pushes previously deposited lignin through the thin network of the primary wall. Thus, lignin is formed in the intercellular spaces. The composition of this lignin differs from the lignin of the S layer, since it was formed earlier and under other conditions (contains fewer methoxyls).

Thus, lignification (the process of hardening and hydrophobization of wood) following the death of cells is caused rather not by the polymerization of lignin, but by the drying of cell wall and the irreversible process of removing hydration shells between carbohydrate macromolecules and the formation of hydrogen bonds between them (Figure 7c,d).

We have developed the structure model of the secondary cell wall (Figure 7b), consisting of cellulose fibers surrounded by lignin–carbohydrate spherical complexes having a carbohydrate shell on the outside and an aromatic core inside. The model also agrees well with the images of cell wall splits (Figure 2).

The isolation of cell vesicles and nanoparticles is a highly valuable strategy to dissect the complex and dynamic nature of the plant transport system affecting cell wall synthesis. We developed a new lignin–carbohydrate vesicle/nanoparticle isolation methodology from aqueous extract of the differentiating xylem and successfully coupled it with different analytical methods to gain insights into the composition of lignin–carbohydrate nano-sized structures. Isolated nanoparticles revealed the presence of cargo destined for the cell wall, including lignin–carbohydrate complexes, highlighting the potential role that nanoparticles play in cell wall synthesis. Adopting this approach nano-sized structures can be isolated, which should provide an increased understanding of cell vesicle functions in different plant pathways.

## 4. Materials and Methods

### 4.1. Materials

The samples of Norway spruce (*Picea abies* (L.) Karst.) growing on the border of its range in Russia, in Arkhangelsk region (64.46° N, 40.94° E.) were harvested. The diameter of the spruce stem was 120 mm. Samples taken in August during the period of active spruce growth. Norway spruce (*Picea abies* (L.) Karst.) was chosen because its xylem is relatively simple anatomically. Furthermore, large-diameter spruce trees were easier to find, allowing us to isolate a thick layer of differentiating xylem. This is important for all types of analysis that require a large sample.

Wheat straw samples (*Triticum* L.) were kindly provided by Altai State University (Barnaul, Russia). The straw content of main components was 21% lignin, 40% cellulose, and 24% hemicellulose. For this study, we also harvested the branches of poplar (Populus balsamifera) trees growing in the University Northern (Arctic) Federal University Arboretum (Russia, Arkhangelsk region, 64°32′ N, 40°33′ E).

The splits of cell wall were prepared from mentioned samples for SEM and AFM studies. The split method with liquid nitrogen was used [2].

### 4.2. Xylem Extract Preparation from Spruce Materials

The spruce stem samples were cut into the blocks. The bark and the secondary phloem layer were carefully removed from the blocks. The differentiating xylem 100 µm thick (slices) was separated and treated by liquid nitrogen. The xylem slices were ground using the MЧ-C mill (Russia) after freezing in liquid nitrogen. Cell walls crack in all directions at this temperature. The xylem material in the form of a powder was stored at −80 °C in freezer (Thermo Fisher Scientific freezer, Marietta, OH, USA). The xylem extract was prepared by adding 1 mL of 0.1 M acetate buffer (pH 5.0) to 100 mg of xylem material with stirring for 1 h. Sodium azide in the amount of 0.05% was added to the suspension to prevent microbial contamination. The xylem extract was separated using centrifugation (Centrifuge 5804R, Eppendorf, Hamburg, Germany) at 14,000 rpm for 1 h at 4 °C to remove all particles larger than 1 μm from the extract (fragments of cell walls, intracellular objects) [34]. Then, the supernatant was treated for ribosomes removing.

Actively functioning cells contain a significant number of ribosomes. Elimination of the interfering effect of ribosomes (mostly 29 nm in diameter) was carried out by treating supernatant of xylem extract with RNase enzyme: the supernatant of xylem extract (10 mL) was mixed with 100 µL RNase solution (RNase A, 17,500 units, QIAGEN). The mixture was incubated for 2 h at room temperature and stirred periodically. Then, the mixture was centrifuged (Centrifuge 5804R, Eppendorf, Germany) for 10 min, at 4200 rpm and room temperature. The supernatant obtained after the treatment was HPLC-SEC separated on several fractions.

### 4.3. Preparative Size-Exclusion Chromatography for Isolation of Lignin-Containing Fraction from the Xylem Extract (HPLC-SEC)

The supernatant fraction containing lignin was isolated from the xylem extract using preparative size-exclusion chromatography Dionex Ultimate 3000 (Thermo Fisher Scientific, Germering, Germany). The volume of the extract was 2 mL. A BioSep-SEC-S4000 preparative column with a pore size of 50 nm was used. 0.1 M acetate buffer (pH 5.0) with 0.05% sodium azide was used as an eluent. The flow rate was 10 mL/min. The detector was UV-280 nm.

The fraction corresponding to the particle sizes from 20 to 50 nm was isolated. This fraction after isolation was frozen at −80 °C (Thermo Fisher Scientific freezer, Marietta, OH, USA) and lyophilized (Labconco FreeZone, Kansas City, MO, USA) as the object for further Py-GC-MS, NMR analysis, and nitrobenzene oxidation.

### 4.4. A Scanning Electron Microscopy

The images were obtained using a scanning electron microscope (SEM) Sigma VP ZEISS (accelerating voltage—10 kV, InLens detector) (Carl Zeiss Microscopy GmbH, Cambridge, UK). To increase the image contrast of the samples while using the SEM, a gold–palladium coating with a thickness of 5 nm was applied. We used Q150 TES device manufactured by QUORUM (Quorum Technologies Ltd., Laughton, UK) for this treatment.

### 4.5. An Atomic Force Microscopy

The images of the samples were obtained at the Multimode 8 Bruker atomic force microscope (Bruker, Santa-Barbara, CA, USA) using a Veeco cantilever (Si-modified Sb) with a rigidity of 62 N/m and an oscillation frequency of 354 kHz. The scan mode was Peak Force.

### 4.6. Pyrolysis Gas Chromatography–Mass Spectrometry (Py-GC-MS)

Py-GC-MS analysis was carried out on a QP-2010Plus gas chromatography—mass spectrometry system (Shimadzu, Kyoto, Japan) equipped with an EGA/PY-3030D pyrolizer (Frontier Lab, Koriyama, Japan) with a liquid nitrogen cooled cryo-trap. The dry samples (1.8–2.7 mg) were placed in stainless steel micro crucibles and subjected to the thermal decomposition in helium atmosphere by the programmed heating from 50 to 600 °C at a rate of 50 °C·min^−1^. The chromatographic separation of the pyrolysis products was achieved on an HP-5ms capillary column (Agilent, Santa Clara, CA, USA) 30 m × 0.25 mm, with a film thickness 0.25 µm. High-purity helium (6.0 grade) with a flow rate of 1 mL·min^−1^ was used as a carrier gas. The column temperature was programmed as follows: 40 °C, held for 2 min; 3 °C·min^−1^ linear ramp to 320 °C, held for 5 min. Mass spectrometry detection was performed with electron ionization; 70 eV in scanning mode; 35–600 Da at a scanning speed of 2 kDa·s^−1^. The detected compounds were identified by the library search NIST/Wiley 2011 databases.

### 4.7. ^1^H NMR Spectroscopy

The ^1^H NMR spectrum was recorded using an AVANCE III 600 NMR spectrometer (Bruker, Ettlingen, Germany) with a resonant frequency for protons of 600 MHz. Bruker Topspin 3.2 software was used for the registration and processing of the experimental data. The sample was dissolved in 400 mL of deuterated water and transferred to a Shigemi microtube. The ^1^H NMR spectrum was recorded at a temperature of 298 K using the zgesgp pulse sequence, which allows suppression of water proton signals (at δH 4.7 ppm); spectral window width—15 ppm, 72 K points, 128 scans, delay—2 s.

### 4.8. Nitrobenzene Oxidation and HPLC-DMD-MS Analysis

Samples of 1 mg were reacted with 0.2 mL of 2 M NaOH solution and 15 µL of nitrobenzene oxidation in a metal autoclave (0.5 mL volume). The autoclave was placed in a muffle furnace and incubated for 2.5 h at 170 °C. The reaction mixture was cooled to room temperature by immersion in running water and then filtered with a glass filter. The filtrate and washings combined were extracted with diethyl ether (2 mL × 3), and the aqueous layer was acidified to 2–3 pH with 1 N HCl solution. Again products were extracted with CH_2_Cl_2_ for 48 h. The extract was evaporated in a nitrogen current. The precipitate (monomeric phenolic products) was dissolved in methanol. Chromatographic separation was carried out on a Zorbax Eclipse Plus C18 column (3 × 150 mm, 3.5 μm, Agilent, USA), at the temperature 40 °C using an LC-30 Nexera HPLC system (Shimadzu, Japan) which consisted of two LC-30AD pumps, a SIL-30A autosampler, a CTO-20A column thermostat, a diode matrix detector CBM-20A. The mobile phase was a mixture of water and methanol (3:1 *v*/*v*) with an admixture of 0.1% HCOOH in the isocratic mode. The flow rate of the mobile phase was 0.6 mL/min. The volume of the injected sample was 10 μL. Monomers were detected using a diode matrix detector in the wavelength range of 250–300 nm [35]. Identification of the target nitrobenzene oxidation products (4-hydroxybenzaldehyde, vanillin, and lilac aldehyde) was performed by comparing the retention times with those of their analytical standards. Mass spectra were recorded on a hybrid QExactive Plus mass spectrometer (Thermo Scientific, Waltham, MA, United States) with an orbitrap mass analyzer with resolution 70,000 FWHM (for *m*/*z* 200) and an IonMax HESI II ion source working in the negative electrospray ionization mode. Mass spectra were recorded in the range *m*/*z* 50–200. The optimum parameters for the ion source in electrospray ionization, ensuring the maximum intensity of the mass spectra of analytes were determined in preliminary experiments as follows: spray voltage 3.2 kV; sheath, aux and sweep gas (N_2_) flow rates 20, 8, and 3 arb. units, respectively; desolvation capillary temperature 320 °C; Aux gas heater temperature 100 °C; and S-lens RF level 55 arb. units. The control of the mass spectrometer, the collection and preprocessing of the data were performed using Xcalibur 4.4 software (Thermo Scientific, Waltham, MA, United States). In the determination of the elemental composition of compounds corresponding to peaks in mass spectra, the admissible relative deviation of the calculated molecular mass from the measured value was taken as 5 ppm. The ratio of syringyl, guaiacyl, and hydroxyphenyl units was determined by comparing the areas of the corresponding chromatographic peaks.

### 4.9. Statistical Analysis of the Results

The qualitative methods for determining the components of lignin–carbohydrate nano-sized structures were used. The scanning electron microscopy has a resolution of 1.3 nm. The similarity index determined by Py-GC-MS analysis is 89–93. The error in determining the molecular weight of the nitrobenzene oxidation products is 0.1 mDa.

SEM visualization has been carried out for a wide range of plant samples: gymnosperms and angiosperms, monocots and dicots. The xylem extract preparation for the experiments requires the extraction of such an amount of biomaterial that definitely leads to the death of the tree. The further research was carried out only with spruce samples as a model object to reduce the environmental damage. This is acceptable, given that a similar pattern of nanoparticles for all plant samples was obtained.

## 5. Conclusions

It has been hypothesized that lignin as a lignin–carbohydrate complex is formed not in the cell wall, but inside the cell. The obtained results are consistent with the assumption that the formation of lignin–carbohydrate complexes occurs in Golgi apparatus and vesicles, which discharged into the inner surface of the cell wall simultaneously with the deposition of cellulose microfibrils. It has been established that lignin–carbohydrate complexes (nanoparticles) have a carbohydrate shell on the outside and an aromatic core inside. A new model of the secondary cell wall structure was proposed based on the results. The model proposes packing of cellulose fibers surrounded by lignin–carbohydrate spherical complexes.

## Figures and Tables

**Table 1:** Products of nitrobenzene oxidation detected using mass spectrometry data.

Products	R_t_, Min	*m*/*z* for [M-H]^−^ Found	*m*/*z* for [M-H]^−^ Calc.	Elemental Composition	Error, ppm	Area	%
4-hydroxybenzaldehyde	3.65	121.0296	121.0295	C_7_H_6_O_2_	1.1	102,130,538	68
Vanillin	4.41	151.0402	151.0400	C_8_H_8_O_3_	1.2	47,822,185	32

## Data Availability

The original contributions presented in this study are included in the article. Further inquiries can be directed to the corresponding author.

## Abbreviations

The following abbreviations are used in this manuscript:

AFM	Atomic force microscopy
HPLC-SEC	Size-exclusion chromatography
LCC	Lignin–carbohydrate complex
Py-GC-MS	Pyrolysis gas chromatography–mass spectrometry
SEM	Scanning electron microscopy
